# The Utility of Sentinel Lymph Node Biopsy in Elderly Patients with Melanoma

**DOI:** 10.1245/s10434-024-15684-0

**Published:** 2024-07-22

**Authors:** Hanna Kakish, Carmen A. Jung, Susan J. Doh, Kathleen M. Mulligan, Iris Sheng, John B. Ammori, Ankit Mangla, Richard S. Hoehn, Luke D. Rothermel

**Affiliations:** 1 Department of Surgery, Division of Surgical Oncology, University Hospitals Cleveland Medical Center, Cleveland, OH USA; 2 Case Western Reserve University School of Medicine, Cleveland, OH USA; 3 Division of Hematology and Oncology, University Hospitals Seidman Cancer Center, Cleveland, OH USA

**Keywords:** Melanoma, Elderly, Sentinel lymph node biopsy, Sentinel lymph node status, Melanoma-specific survival, Overall survival

## Abstract

**Background:**

Sentinel lymph node biopsy (SLNB) is performed less often for older patients with melanoma. We investigated the association of SLNB and melanoma-specific survival (MSS) in the elderly.

**Methods:**

We retrospectively reviewed the Surveillance, Epidemiology, and End Results (SEER: 2010–2019) for patients ≥ 70 years with cT2-4N0M0 melanoma. We used multivariable Cox proportional hazard models to evaluate the impact of SLNB performance and SLN status on MSS at increasing age cutoffs. In addition, we evaluated the association of different factors with SLNB performance using multivariable logistic regression.

**Results:**

We identified 11,548 patients. Sentinel lymph node biopsy occurred in 6754 (58.5%) patients, 1050 (15.5%) of whom had a positive SLN. On adjusted SEER analysis, a negative SLN was independently associated with improved MSS (overall hazard ratio [HR] 0.59, 95% confidence interval [CI] 0.63–0.67) for patients up to 87 years old. Positive SLNB was independently associated with inferior MSS (HR 1.71, 95% CI 1.93–1.98). Increasing age groups were significantly associated with decreased SLNB performance.

**Conclusions:**

Sentinel lymph node biopsy is associated with cancer-specific survival and adds prognostic information for elderly patients with melanoma. Sentinel lymph node biopsy performance should not be eliminated in elderly patients based on age alone, unless justified by poor performance status, patient preference, or other surgical contraindications. Decreased SLNB performance with increasing age in our cohort may indicate a missed therapeutic opportunity in the care of elderly patients with melanoma.

**Supplementary Information:**

The online version contains supplementary material available at 10.1245/s10434-024-15684-0.

The National Comprehensive Cancer Network (NCCN) recommends sentinel lymph node biopsy (SLNB) for patients with melanoma >1.0 mm in Breslow depth.^1^ Despite having an increased incidence of melanoma, elderly patients are less likely to undergo SLNB.^2,3^ This may be attributed to patient frailty, medical comorbidities, or provider perceptions that sentinel lymph node (SLN) status bears less importance than in younger populations.^4–7^ The prognostic and therapeutic benefits of SLNB in elderly patients with melanoma are debated.^8–10^

The results of the Multicenter Selective Lymphadenectomy Trial-I (MSLT-I) trial showed that performing SLNB decreased regional nodal recurrences but did not impact melanoma-specific survival (MSS) for the overall cohort of patients with intermediate and thick melanomas.^8,11^ However, the authors acknowledged that low nodal positivity rates in this cohort may have blunted the MSS benefit.^8^ In addition, patients older than 75 years were not enrolled in the MSLT-I trial. Hence, the utility of SLNB remains debatable in elderly patients who more often present with other comorbidities. Murtha et al. studied the Surveillance, Epidemiology, and End Results (SEER) database (2010–2012) and reported an improved association of MSS in patients with intermediate and thick melanomas who underwent SLNB across all age groups.^9^ Sabel et al. performed an institutional study of 553 melanoma (> 1.0-mm Breslow depth) patients ≥ 75 years between 1996 and 2011 and reported lower MSS for patients who did not undergo SLNB, yet this result did not reach statistical significance (*p* = 0.172).^10^ The question remains whether SLNB adds value for elderly patients with melanoma when considering the risks and benefits of the procedure.

The primary objective of this study was to evaluate the association of SLNB and MSS in elderly patients (≥ 70 years) with melanoma using a national dataset (SEER) with the ability to assess cancer-specific survival. We anticipated that if a survival benefit was lost or diminished in the elderly population, this would argue against the prognostic utility of SLNB in elderly patients. Secondarily, we assessed factors associated with SLNB performance and SLN positivity. We hypothesized that SLNB will provide similar benefits in the elderly to those seen in the broader population and that the procedure remains warranted in elderly patients who are fit to undergo surgery.

## Materials and methods

### Data Source and Institutional Review Board Exemption

This is a retrospective study of patients with invasive cutaneous melanoma using the SEER database. The data used in this project is publicly available and deidentified; therefore, this study is exempt from IRB review and approval as determined by the University Hospitals Cleveland Medical Center IRB (STUDY20231391). The SEER database is a jointly issued (by the ACS, the Centers for Disease Control and Prevention (CDC), the North American Association of Central Cancer Registries (NAACCR), and the NCI) cancer registry,^12^ which represents approximately 48% of the U.S. population from 19 geographic areas.^13^ We utilized the Incidence–SEER Research Plus Data, 17 Registries, Nov 2021 Sub (2000–2019) database, which was based on data submitted from November 2021.

### Patient Selection

**Fig. 1:**
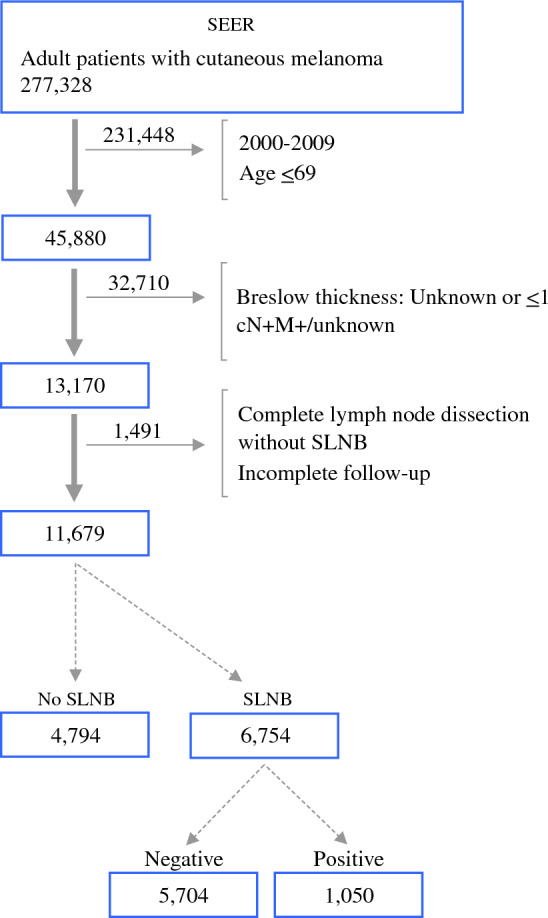
Inclusion criteria

We identified patients with the diagnosis of cutaneous melanoma (2010–2019) by using the International Classification of Disease (ICD) codes (ICD-O-3: C44.0-C44.9) and histology codes (8720-8723, 8730, 8740-8745, 8761, and 8770-8774). We extracted cases for patients ≥ 70 years diagnosed with cutaneous melanoma > 1 mm in Breslow depth. To minimize the chance that thicker melanomas could be a confounding factor in our analysis of intermediate-thickness melanomas, we excluded patients with tumors ≤ 4.0 mm in Breslow depth when the deep margin was transected at biopsy. Furthermore, we excluded patients with distant or regional lymph node metastasis, those who underwent lymph node aspiration or complete lymph node dissection without SLNB, and those with unknown survival data and SLN status. Finally, we excluded patients for whom melanoma was not the primary listed tumor among patients registered with multiple tumors. Figure 1 depicts the inclusion flowchart.

### Study Variables

We collected data for baseline demographics (age, sex, and race), tumor characteristics (SLNB performance, nodal positivity, tumor location, tumor subtype, ulceration status, and mitotic rate), and receipt of adjuvant treatment. Patient age at diagnosis was divided into groups 70–79, 80–89, ≥ 90 years. Race/ethnicity categories included non-Hispanic White and others (non-Hispanic Black, Asian, Hispanic, and others/unknown). Tumor locations were designated head and neck, upper extremity, lower extremity, trunk, overlapping lesions, or not specified. Overlapping lesions have overlapping boundaries of two or more categories or subcategories. Histologic subtypes included nodular, lentigo maligna, superficial spreading, acral lentiginous, desmoplastic, and others. Mitotic rate was classified as: nonmitogenic, 1 mitotic figure/mm^2^, ≥ 2 mitotic figures/mm^2^, and other/unknown. In addition, we included presence and absence of ulceration. Both variables “RX Summ—Scope Reg LN Sur” and “Regional nodes positive” were used to identify patients who underwent SLNB with or without LN involvement.^14^ We included patients registered in 2010 or after because certain variables such as mitotic rate were not collected before then.

### Statistical Analysis

We stratified our cohort into two groups based on SLNB performance (patients who underwent SLNB vs. those who did not), and those who underwent SLNB based on SLN results (positive: sentinel lymph node involved with the disease vs. negative: sentinel lymph node not involved with the disease). Categorical variables were compared by using Pearson’s chi-square test, and multivariable logistic regression was utilized to assess the association between different factors and SLNB performance or SLN metastasis. We reported the 5-year MSS between patients who underwent SLNB versus those who did not undergo SLNB. Next, because we noticed a survival difference between the previous groups (SLNB vs. no SLNB), we reported the 5-year MSS between patients who underwent SLNB with positive and negative results compared with those who did not undergo SLNB. Next, we studied the association between SLNB and MSS (groups: SLN positive, SLN negative, and no SLNB) in elderly melanoma patients overall (≥ 70 years) and at increasing age cutoffs (≥ 71, ≥ 72, ≥ 73, ≥ 74…, ≥ 90) (age removed from these models) by using multivariable Cox proportional hazard models. We performed our analysis at increasing age cutoffs to identify if the association between performing SLNB and MSS, if present, persisted in more elderly patients. Similarly, we performed a similar analysis for patients with T2–3 and T4 tumors separately. We performed all statistical analyses using Stata 17^®^, defining statistical significance as *p* < 0.05.^15^

### Subgroup Analysis

The question of whether SLNB is associated with improved MSS in intermediate and thick melanomas remains controversial across all age groups. Therefore, as a comparison, we studied the association between SLNB and MSS in melanoma patients < 70 years at increasing age cutoffs (≥ 21, ≥ 22, ≥ 23, ≥ 24…, ≥ 69) (age removed from these models) using multivariable Cox proportional hazard models.

## Results

### Study Cohort

Table 1 shows the characteristics of patients in our SEER cohort. Our analysis identified 11,548 patients who fit our inclusion criteria, 6,754 (58.5%) of whom underwent SLNB. For those who underwent SLNB, 1,050 (15.5%) patients had SLN metastasis and 5,704 (84.5%) patients did not have SLN metastasis. Sentinel lymph node biopsy performance decreased significantly with age (70–79 years = 72.8%, 80–89 years = 48.7%, and ≥ 90 years = 16%, *p* < 0.001). However, our analysis did not show a significant difference in the rate of SLN positivity among the different age groups (14.5–16.8%, *p* > 0.05) for those who underwent a SLNB.

**Table 1:** Summary of patients ≥ 70 years based on SLNB in the Surveillance, Epidemiology, and End Results database (2010 to 2019) diagnosed with clinically nonmetastatic melanomas >1.0 mm in Breslow thickness

	No SLNB	SLNB	*p*	SLN negative	SLN-positive	*p*
Total	4794	6754		5704 (84.5)	1050 (15.5)	
Age (years)						
70–79	1688 (27.2)	4519 (72.8)	< 0.001	3797 (84)	722 (16)	0.280
80–89	2167 (51.3)	2056 (48.7)		1758 (85.5)	298 (14.5)	
≥ 90	939 (84)	179 (16)		149 (83.2)	30 (16.8)	
Sex						
Male	2851 (39.7)	4338 (60.3)	< 0.001	3672 (84.6)	666 (15.4)	0.560
Female	1943 (44.6)	2416 (55.4)		2032 (84.1)	384 (15.9)	
Race						
Non-Hispanic White	4445 (41)	6403 (59)	< 0.001	5440 (85)	963 (15)	< 0.001
Others	349 (49.9)	351 (50.1)		264 (75.2)	87 (24.8)	
Tumor location						
Head/neck	1988 (54.7)	1647 (45.3)	< 0.001	1488 (90.3)	159 (9.7)	< 0.001
Trunk	932 (36.5)	1618 (63.5)		1304 (80.6)	314 (19.4)	
Upper limb/shoulder	1122 (33.8)	2195 (66.2)		1913 (87.2)	282 (12.8)	
Lower limb/hip	728 (36.4)	1274 (63.6)		983 (77.2)	291 (22.8)	
Others	24 (54.5)	20 (45.5)		16 (80)	4 (20)	
Tumor subtype						
Nodular	1137 (41.3)	1614 (58.7)	< 0.001	1281 (79.4)	333 (20.6)	< 0.001
Lentigo maligna	245 (51.6)	230 (48.4)		217 (94.3)	13 (5.7)	
Superficial spreading	865 (36.5)	1502 (63.5)		1272 (84.7)	230 (15.3)	
Acral lentiginous	91 (29.9)	213 (70.1)		142 (66.7)	71 (33.3)	
Desmoplastic	236 (47.7)	259 (52.3)		246 (95)	13 (5)	
Others	2220 (43.1)	2936 (56.9)		2546 (86.7)	390 (13.3)	
Breslow thickness						
>1.0–2.0	1919 (39.2)	2977 (60.8)	< 0.001	2682 (90.1)	295 (9.9)	< 0.001
>2.0–4.0	1469 (39.9)	2211 (60.1)		1866 (84.4)	345 (15.6)	
>4.0	1406 (47.3)	1566 (52.7)		1156 (73.8)	410 (26.2)	
Ulceration						
No	2746 (40.5)	4032 (59.5)	0.020	3545 (87.9)	487 (12.1)	< 0.001
Yes	1967 (43.1)	2598 (56.9)		2044 (78.7)	554 (21.3)	
Unknown	81 (39.5)	124 (60.5)		115 (92.7)	9 (7.3)	
Mitosis						
Nonmitogenic	548 (44.8)	674 (55.2)	< 0.001	628 (93.2)	46 (6.8)	< 0.001
1 mitotic figure/mm^2^	652 (40.3)	964 (59.7)		854 (88.6)	110 (11.4)	
≥ 2 mitotic figures/mm^2^	2855 (39.4)	4394 (60.6)		3602 (82)	792 (18)	
Others/unknown	739 (50.6)	722 (49.4)		620 (85.9)	102 (14.1)	
County				N/A		
Metropolitan	4059 (40.8)	5887 (59.2)	< 0.001			
Non-metropolitan	734 (45.9)	865 (54.1)				
Income				N/A		
< $50,000	767 (48.5)	813 (51.5)	< 0.001			
$50,000–$74,999	2458 (40.1)	3670 (59.9)				
≥ $75,000	1568 (40.8)	2271 (59.2)				

*SLNB* sentinel lymph node biopsy; *SLN* sentinel lymph node

### Predictors of SLNB Performance and SLN Status

On multivariable analysis for predictors of SLNB performance (Table 2), tumors that were mitotic, acral lentiginous (vs. superficial spreading), and present on the extremity (vs. trunk) had significantly higher likelihood of undergoing SLNB. Our analysis reported lower likelihood of SLNB performance with increased age, female sex, races other than non-Hispanic White, head and neck tumors, lentigo maligna subtype, and Breslow depth >4.0 mm (compared with >1–2 mm tumors). Furthermore, SLNB performance was more likely in patients with higher income. On adjusted analysis for nodal positivity (Table 2), increased likelihood of nodal positivity was seen with acral lentiginous melanomas (vs. superficial spreading), increased Breslow thickness, ulceration, and mitotic tumors. In contrast, nodal positivity was less likely in patients 80–89 years (vs. 70–79), tumors on the head and neck and upper extremity (vs. trunk), and lentigo maligna and desmoplastic melanomas (vs. superficial spreading).

**Table 2:** Predictors of SLNB performance and sentinel lymph node positivity in the Surveillance, Epidemiology, and End Results database (2010–2019) for patients diagnosed with clinically nonmetastatic melanomas >1.0 mm in Breslow thickness

	SLNB performance		SLN positivity	
	Odds ratio	95% CI	Odds ratio	95% CI
Age (ref = 70–79)				
80–89	0.35	0.32–0.39	0.82	0.7–0.95
≥ 90	0.07	0.06–0.08	0.95	0.62–1.44
Sex (ref = male)				
Female	0.81	0.74–0.89	0.94	0.81–1.1
Race (ref = non-Hispanic White)		–		
Others	0.62	0.52–0.74	1.30	0.99–1.72
Tumor location (ref = trunk)				
Head/neck	0.55	0.49–0.61	0.48	0.39–0.59
Upper limb/shoulder	1.38	1.23–1.56	0.61	0.51–0.73
Lower limb/hip	1.29	1.11–1.48	1.07	0.87–1.32
Others	0.53	0.27–1.02	0.99	0.3–3.22
Tumor subtype (ref = superficial spreading)				
Nodular	0.98	0.86–1.12	0.97	0.79–1.18
Lentigo maligna	0.78	0.63–0.98	0.48	0.27–0.86
Acral lentiginous	1.39	1.03–1.88	1.51	1.05–2.17
Desmoplastic	0.97	0.77–1.21	0.26	0.14–0.47
Others	0.88	0.78–0.98	0.73	0.6–0.87
Breslow thickness (ref = >1.0–2.0)				
>2.0–4.0	1.04	0.94–1.16	1.43	1.19–1.71
>4.0	0.8	0.71–0.9	2.71	2.24–3.28
Ulceration (ref = no)				
Yes	1.01	0.92–1.11	1.32	1.14–1.53
Mitosis (ref = nonmitogenic)		-		
1 mitotic figure/mm^2^	1.23	1.05–1.45	1.65	1.14–2.38
≥ 2 mitotic figures/mm^2^	1.48	1.29–1.71	2.1	1.52–2.91
Others/unknown	0.89	0.75–1.06	2.13	1.46–3.12
County (ref = metropolitan)			N/A	
Nonmetropolitan	0.89	0.77–1.02		
Income (ref = <$50,000)			N/A	
$50,000–$74,999	1.46	1.27–1.68		
≥ $75,000	1.44	1.24–1.68		

*SLNB* sentinel lymph node biopsy; *SLN* sentinel lymph node; *CI* confidence interval

### Survival

**Fig. 2:**
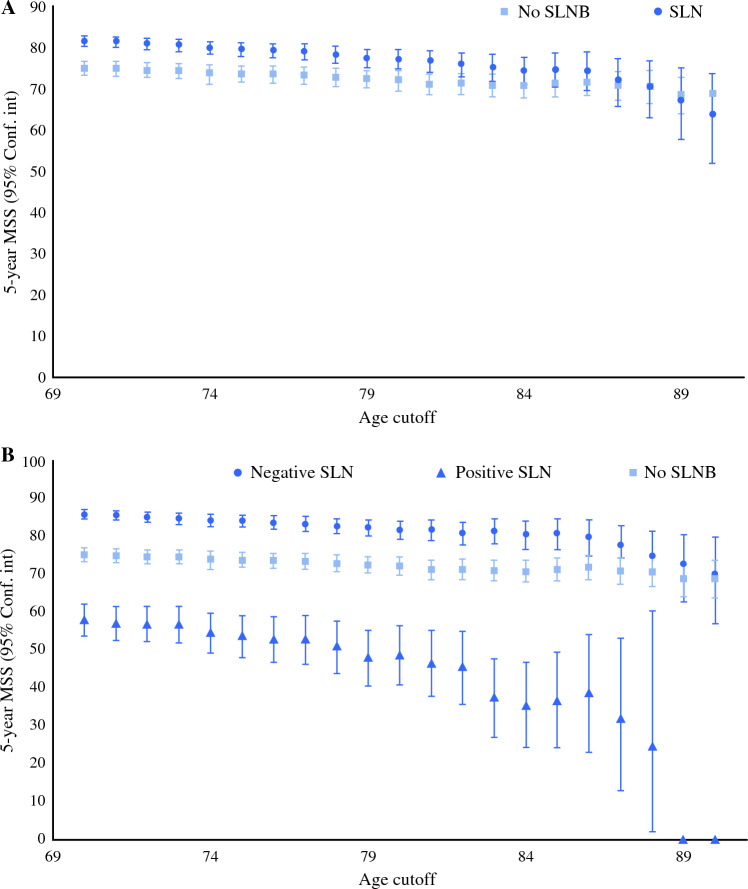
Five-year melanoma-specific survival based on SLNB for patients with clinically nonmetastatic melanomas >1.0 mm in Breslow thickness in the Surveillance, Epidemiology, and End Results database (2010–2019) (**A**: SLNB vs. no SLNB, **B**: no SLNB vs. negative SLN vs. positive SLN)

**Fig. 3:**
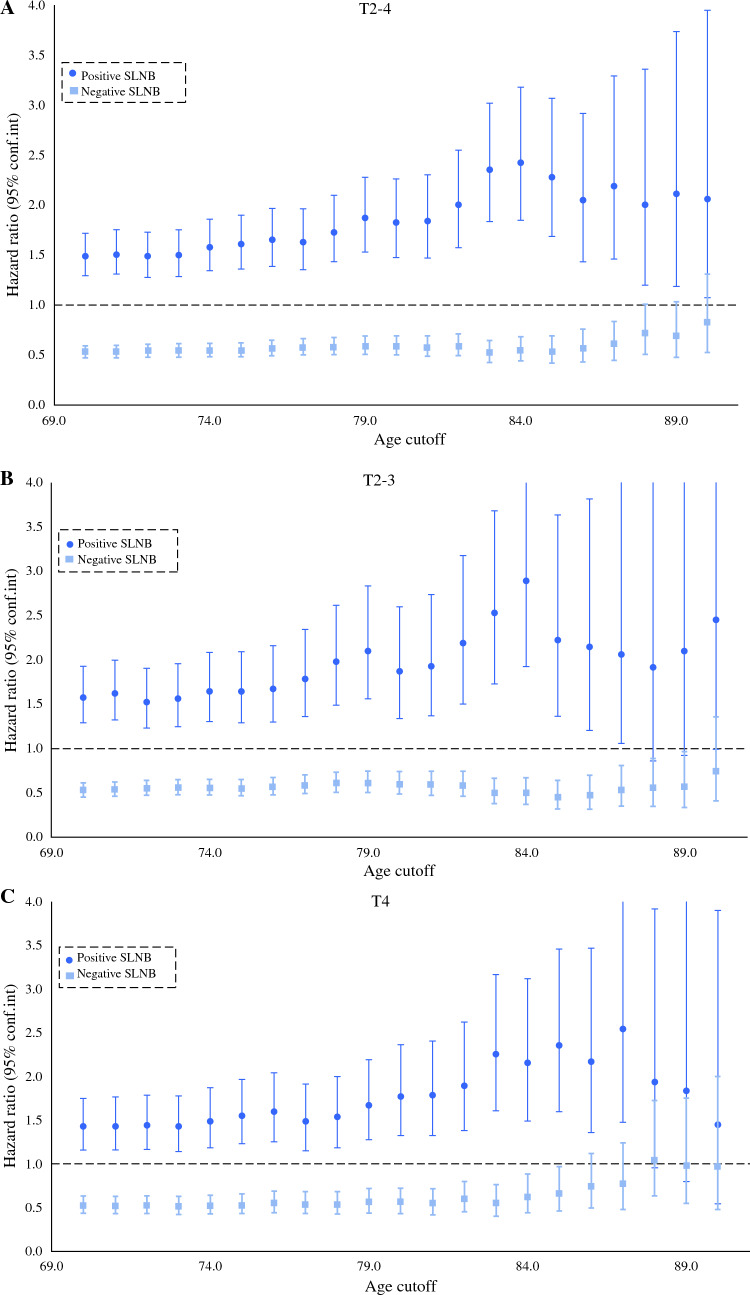
Multivariable Cox proportional hazard model reporting hazard ratios for the effect of SLNB on melanoma-specific survival using SEER (2010–2019) at increasing age cutoffs (starting at ≥ 70) for patients diagnosed with clinically nonmetastatic melanomas >1.0 mm in Breslow thickness (overall cohort [**A**: T2–4] and separated by T-stage [**B**: T2–3 and **C**: T4])

Kaplan–Meier survival analysis identified an increased 5-year MSS for patients who underwent SLNB (81.7%, 95% confidence interval [CI] 80.5–82.9%) compared with those who did not undergo SLNB (75.1%, 95% CI 73.3–76.8%), *p* < 0.001. The difference in MSS was significant up to 86-year-old patients. Next, we separated patients who underwent SLNB into negative SLN and positive SLN. Our analysis reported increased 5-year MSS for patients with negative SLN (85.7%, 95% CI 84.5–86.9%) compared with those who did not undergo SLNB and those with a positive SLN (58%, 95% CI 53.7–62.2%), *p* < 0.001. This result were consistent across the entire age continuum (Fig. 2). Multivariable Cox analysis identified an association between negative SLN and improved MSS (compared with patients who did not undergo SLNB, hazard ratio [HR] 0.59; 95% CI 0.63–0.67) (Supplemental Table 1). Inferior MSS was associated with increased age, positive SLN, male sex, acral lentiginous subtype, increased Breslow thickness, ulcerated tumors, and ≥ 2 mitotic figures/mm^2^. Next, we performed multiple regression models (removing the age variable) to study the effect of SLNB at increasing age cutoffs (Fig. 3). The association between negative SLN and improved MSS (compared with no SLNB) remained significant for patients at increasing age cutoffs until age ≥ 87 years. No significant association between negative SLN and MSS was seen in age ≥ 88 years; however, low event rates in older age cohorts may limit the accuracy of this data. When this data was stratified by intermediate (T2–3) versus thick (T4) melanomas, the impact of negative SLN on MSS was less for the T4 tumors. Multivariable Cox analysis showed positive SLN to be significantly associated with worse MSS (compared with no SLNB) at increasing age cutoffs across all age cutoffs.

### Subgroup Analysis

Multivariable Cox analysis of patients ≤69 years old identified similar association between negative SLN and improved MSS in the overall cohort and when separated by T-stage compared with patients not undergoing SLNB (Supplemental Fig. 1).

## Discussion

The incidence of melanoma in the geriatric population is increasing at a faster rate than in younger patients.^2,16^ Increasing age also is associated with primary melanomas exhibiting worse features (such as increased Breslow thickness, ulceration, etc.) and a higher incidence of melanoma-related deaths.^17–19^ However, the existing literature is inconsistent about the importance of SLNB in the elderly. Sentinel lymph node biopsy is used in the general population as an important prognostic tool that informs treatment decisions and imaging surveillance for qualified melanoma patients. Importantly, systemic treatments, such as immune and targeted therapies, used for patients with melanoma are tolerable and beneficial even in elderly populations.^20^ Therefore, while acknowledging that prospective studies are needed to confirm the association between SLNB performance and MSS, our data identified an association between SLNB and MSS, persisting for most elderly patients.

In this analysis, SLNB utilization decreased from >70% in patients aged 70–79 years to <50% for patients 80 years or older. Lower SLNB utilization in elderly patients is likely driven by multiple factors involving patient, tumor, and procedural considerations. The surgical complication rate of 5–11%, the vast majority of which are temporary and not lifestyle-limiting, may dissuade providers from performing the procedure in frail patients.^21,22^ In addition, elderly patients are at higher risk of perioperative morbidity and mortality, including anesthesia-related complications, such as cognitive dysfunction.^23^ Furthermore, elderly patients exhibit altered lymphatic drainage, as evidenced by poorer uptake of radiotracer dye during lymphoscintigraphy,^24,25^ and their tumors may have higher rates of hematogenous spread.^17,26^ These anatomic and biologic differences may influence a provider’s perception of value regarding SLNB. Nonetheless, our data describe a reasonably high and consistent rate of nodal positivity (15–18%) across elderly age groups. Whereas it is sensible to consider the perioperative risks of SLNB in this population, differences in anatomic and biologic factors in the elderly do not preclude the prognostic value of the procedure.

There may be other benefits to identifying occult nodal disease for elderly patients. A latent-subgroup analysis of the MSLT-I reported significantly improved MSS (HR = 0.68; *p* = 0.05) and distant metastasis-free survival (HR = 0.73; *p* = 0.04) for patients with intermediate-thickness tumors in whom node-positive disease was discovered on SLNB rather than after a clinical recurrence.^8^ Moreover, nodal recurrences are associated with worse quality of life and an increased risk of long-term morbidity after complete lymph node dissection.^27^ MSLT-I data indicate that essentially all metastases detected by SLNB would have eventually become clinically evident if not removed, as there were no differences in the rate of nodal metastasis identified between the SLNB and the observation group with long-term follow-up. While the national dataset used in this study cannot determine rates of melanoma recurrence, performing a SLNB has been shown to consistently decrease rates of regional recurrence in large randomized controlled trials regardless of tumor thickness in nonelderly patients (≤75 years).^8,28^ Whether elderly patients with occult positive nodes identified by SLNB fare better than those with nodal recurrences after observation requires further study with more granular longitudinal data.

The finding of improved MSS for patients undergoing SLNB, which also was seen in the younger cohort, was surprising. There are rationales that could explain the improved MSS for patients undergoing a SLNB. For instance, the *incubator* theory for cancer progression suggests that metastasis to a lymph node is essential for the priming of melanoma cells to survive as distant metastases.^29^ Preclinical studies have suggested a mechanism for this priming through the process of ferroptosis within lymph nodes.^30^ If this theory is correct, removal of draining lymph nodes before melanoma cells are primed for distant metastasis could improve MSS and may explain the findings in this large retrospective study. However, as mentioned previously, no prospective data has definitively shown an association between SLNB and improved MSS. It may be more likely that our results are influenced by a selection bias wherein more high-risk patients are foregoing SLNB in the elderly group, which may worsen the prognosis of the cohort not undergoing SLNB. While we cannot conclude that there is a definitive association between SLNB performance and MSS based on this retrospective study, there appears to be a potential benefit in younger age groups that persists in elderly patients, using the SEER database.

There are several limitations when interpreting data from this study. The retrospective nature of the study introduces selection biases. The lack of detailed information in a deidentified national dataset could obscure confounding factors. For instance, we were unable to account for the false-negative rate for patients who underwent SLNB, weigh the risks and benefits of SLNB, or include other outcomes such as recurrence-free survival. Also, not all factors that influence the decision for or against SLNB are captured in this dataset. Examples of this include frailty, inability to undergo general anesthesia, patients’ preference (with concern about prolonging recovery), physician recommendations, other comorbidities, proximity to treatment providers, and the limited life expectancy in the extremely elderly patients who are vulnerable to poorer outcomes even if fewer comorbidities are listed.^3^ The SEER program does not recommend comparing outcomes conditioned on systemic treatment using the SEER data without careful considerations of possible biases and appropriate adjustments. In addition, during the time period studied, adjuvant therapies for melanoma were variable, and utilization of contemporary treatments affects care substantially. Therefore, our study lacks sufficient information to suggest whether these therapies impact MSS, and the relevance of this data remains in question. Finally, although we used SEER to determine melanoma-specific survival, the accuracy of the “cause-specific survival” variable in the SEER database is debated. Cancer registries use algorithms to capture the cause of death from death certificates and identifying a single cause may be difficult, leading to a possible over- or under-attribution of cancer as a cause of death.^31^ For example, a death may be attributed to a site of metastasis or to a side effect of treatment. Although these deaths are not cancer deaths in a biological sense, they reflect the consequences of cancer. In addition, the cancer-specific survival becomes less reliable in elderly patients, raising inaccuracies as well.^32^ Although we interpret our results with caution, several authors have used a similar methodology and determined that using the SEER database’s cause-specific survival in calculating cancer-specific survival has acceptable validity.^33^

## Conclusions

This analysis of the SEER database shows a prognostic benefit to SLNB in elderly patients and suggests a persistent association between SLNB performance and SLN status and melanoma-specific survival in all age groups. The survival benefit was neither eliminated nor decreased in the elderly population. Whereas some elderly patients may not undergo SLNB because of poor performance status, patient preference, or contraindications to surgery, our analysis highlights that SLNB should not be omitted based on age alone if patients can otherwise tolerate surgery.

## Supplementary Information

Below is the link to the electronic supplementary material.

Supplementary file1 (TIFF 260 kb)

Supplementary file2 (DOCX 17 kb)

## References

[1] National Comprehensive Cancer Network. Melanoma (Version3.2022).www.nccn.org/patients. Accessed 1 Oct 2022.

[2] Paulson KG, Gupta D, Kim TS, et al. Age-specific incidence of melanoma in the United States. *JAMA Dermatol*. 2020;156(1):57. 10.1001/jamadermatol.2019.3353. 31721989 10.1001/jamadermatol.2019.3353 PMC6865303

[3] Rees MJ, Liao H, Spillane J, et al. Melanoma in the very elderly, management in patients 85 years of age and over. *J Geriatr Oncol*. 2018;9(5):488–93. 10.1016/j.jgo.2018.01.001. 29398454 10.1016/j.jgo.2018.01.001

[4] Koskivuo I, Hernberg M, Vihinen P, et al. Sentinel lymph node biopsy and survival in elderly patients with cutaneous melanoma. *Br J Surg*. 2011;98(10):1400–7. 10.1002/bjs.7565. 21638276 10.1002/bjs.7565

[5] Cavanaugh-Hussey MW, Mu EW, Kang S, Balch CM, Wang T. Older age is associated with a higher incidence of melanoma death but a lower incidence of sentinel lymph node metastasis in the SEER databases (2003–2011). *Ann Surg Oncol*. 2015;22(7):2120–6. 10.1245/s10434-015-4538-8. 25940571 10.1245/s10434-015-4538-8

[6] Macdonald JB, Dueck AC, Gray RJ, et al. Malignant melanoma in the elderly: different regional disease and poorer prognosis. *J Cancer*. 2011;2:538–43. 10.7150/jca.2.538. 22084644 10.7150/jca.2.538 PMC3213678

[7] Featherston C, Nardi WS, Tomé FR, Quildrian SD. Role of sentinel lymph node biopsy for cutaneous melanoma in elderly patients: preliminary results in a Latin-American population. *Ecancermedicalscience*. 2021;15:1167. 10.3332/ecancer.2021.1167. 33680081 10.3332/ecancer.2021.1167 PMC7929767

[8] Morton DL, Thompson JF, Cochran AJ, et al. Final trial report of sentinel-node biopsy versus nodal observation in Melanoma. *N Engl J Med*. 2014;370(7):599–609. 10.1056/NEJMoa1310460. 24521106 10.1056/NEJMoa1310460 PMC4058881

[9] Murtha TD, Han G, Han D. Predictors for use of sentinel node biopsy and the association with improved survival in melanoma patients who have nodal staging. *Ann Surg Oncol*. 2018;25(4):903–11. 10.1245/s10434-018-6348-2. 29368153 10.1245/s10434-018-6348-2

[10] Sabel MS, Kozminski D, Griffith K, Chang AE, Johnson TM, Wong S. Sentinel lymph node biopsy use among melanoma patients 75 years of age and older. *Ann Surg Oncol*. 2015;22(7):2112–9. 10.1245/s10434-015-4539-7. 25834993 10.1245/s10434-015-4539-7

[11] Multicenter Selective Lymphadenectomy Trials Study Group, Crystal JS, Thompson JF, et al. Therapeutic value of sentinel lymph node biopsy in patients with melanoma: a randomized clinical trial. *JAMA Surg*. 2022;157(9):835-42. 10.1001/jamasurg.2022.2055 10.1001/jamasurg.2022.2055 PMC9475390 35921122

[12] Surveillance, Epidemiology, and End Results (SEER) Program (www.seer.cancer.gov) SEER*Stat Database: Incidence - SEER Research Plus Data, 17 Registries, Nov 2021 Sub (2000-2019) - Linked To County Attributes - Time Dependent (1990-2019) Income/Rurality, 1969–2020 Counties, National Cancer Institute, DCCPS, Surveillance Research Program, released April 2022, based on the November 2021 submission.

[13] SRP Communications N. *Surveillance Research Program (SRP) Surveillance, Epidemiology and End Results (SEER) Program Metrics*. 2020.

[14] *Adamo M, Groves C, Dickie L, Ruhl J. (September 2022). SEER Program Coding and Staging Manual 2023. National Cancer Institute, Bethesda, MD 20892. U.S. Department of Health and Human Services National Institutes of Health National Cancer Institute*.

[15] StataCorp. Stata Statistical Software: Release 17. College Station, TX: StataCorp LLC; 2021.

[16] Thrift AP, Gudenkauf FJ. Melanoma incidence among non-hispanic whites in all 50 US States from 2001 through 2015. *JNCI*. 2020;112(5):533–9. 10.1093/jnci/djz153. 31346623 10.1093/jnci/djz153 PMC7225671

[17] Chao C, Martin RCG, Ross MI, et al. Correlation between prognostic factors and increasing age in melanoma. *Ann Surg Oncol*. 2004;11(3):259–64. 10.1245/aso.2004.04.015. 14993020 10.1245/aso.2004.04.015

[18] Balch CM, Thompson JF, Gershenwald JE, et al. Age as a predictor of sentinel node metastasis among patients with localized melanoma: an inverse correlation of melanoma mortality and incidence of sentinel node metastasis among young and old patients. *Ann Surg Oncol*. 2014;21(4):1075–81. 10.1245/s10434-013-3464-x. 24531700 10.1245/s10434-013-3464-x PMC4121329

[19] Lasithiotakis K, Leiter U, Meier F, et al. Age and gender are significant independent predictors of survival in primary cutaneous melanoma. *Cancer*. 2008;112(8):1795–804. 10.1002/cncr.23359. 18306371 10.1002/cncr.23359

[20] Bastiaannet E, Battisti N, Loh KP, et al. Immunotherapy and targeted therapies in older patients with advanced melanoma; Young International Society of Geriatric Oncology review paper. *J Geriatr Oncol*. 2019;10(3):389–97. 10.1016/j.jgo.2018.06.009. 30025821 10.1016/j.jgo.2018.06.009 PMC8074511

[21] Moody JA, Ali RF, Carbone AC, Singh S, Hardwicke JT. Complications of sentinel lymph node biopsy for melanoma—A systematic review of the literature. *Eur J Surg Oncol*. 2017;43(2):270–7. 10.1016/j.ejso.2016.06.407. 27423448 10.1016/j.ejso.2016.06.407

[22] Brănişteanu DE, Cozmin M, Porumb-Andrese E, et al. Sentinel lymph node biopsy in cutaneous melanoma, a clinical point of view. *Medicina (Kaunas)*. 2022. 10.3390/medicina58111589. 36363546 10.3390/medicina58111589 PMC9698247

[23] Staheli B, Rondeau B. *Anesthetic Considerations in the Geriatric Population*. 2024. 34283503

[24] Castle SC, Uyemura K, Fulop T, Makinodan T. Host resistance and immune responses in advanced age. *Clin Geriatr Med*. 2007;23(3):463–79. 10.1016/j.cger.2007.03.005. 17631228 10.1016/j.cger.2007.03.005 PMC7135540

[25] Conway WC, Faries MB, Nicholl MB, et al. Age-related lymphatic dysfunction in melanoma patients. *Ann Surg Oncol*. 2009;16(6):1548–52. 10.1245/s10434-009-0420-x. 19277787 10.1245/s10434-009-0420-x PMC2752947

[26] Sasson DC, Smetona JT, Parsaei Y, et al. Malignant melanoma in older adults: different patient or different disease? *Cureus*. 2023;15(2):e34742. 10.7759/cureus.34742. 36909026 10.7759/cureus.34742 PMC9998075

[27] Faries MB, Thompson JF, Cochran A, et al. The impact on morbidity and length of stay of early versus delayed complete lymphadenectomy in melanoma: results of the multicenter selective lymphadenectomy trial (I). *Ann Surg Oncol*. 2010;17(12):3324–9. 10.1245/s10434-010-1203-0. 20614193 10.1245/s10434-010-1203-0 PMC2970739

[28] Leiter U, Stadler R, Mauch C, et al. Final analysis of DeCOG-SLT trial: No survival benefit for complete lymph node dissection in patients with melanoma with positive sentinel node. *J Clin Oncol*. 2019;37(32):3000–8. 10.1200/JCO.18.02306. 31557067 10.1200/JCO.18.02306

[29] Faries MB, Han D, Reintgen M, Kerivan L, Reintgen D, Caracò C. Lymph node metastasis in melanoma: a debate on the significance of nodal metastases, conditional survival analysis and clinical trials. *Clin Exp Metastasis*. 2018;35(5–6):431–42. 10.1007/s10585-018-9898-6. 29777421 10.1007/s10585-018-9898-6 PMC6202130

[30] Ubellacker JM, Tasdogan A, Ramesh V, et al. Lymph protects metastasizing melanoma cells from ferroptosis. *Nature*. 2020;585(7823):113–8. 10.1038/s41586-020-2623-z. 32814895 10.1038/s41586-020-2623-z PMC7484468

[31] Howlader N, Ries LAG, Mariotto AB, Reichman ME, Ruhl J, Cronin KA. Improved estimates of cancer-specific survival rates from population-based data. *J Natl Cancer Inst*. 2010;102(20):1584–98. 10.1093/jnci/djq366. 20937991 10.1093/jnci/djq366 PMC2957430

[32] Forjaz de Lacerda G, Howlader N, Mariotto AB. Differences in cancer survival with relative versus cause-specific approaches: an update using more accurate life tables. *Cancer Epidemiol Biomarkers Prev*. 2019;28(9):1544-51. 10.1158/1055-9965.EPI-19-0125 10.1158/1055-9965.EPI-19-0125 PMC6726514 31221717

[33] Hu CY, Xing Y, Cormier JN, Chang GJ. Assessing the utility of cancer-registry-processed cause of death in calculating cancer-specific survival. *Cancer*. 2013;119(10):1900–7. 10.1002/cncr.27968. 23408226 10.1002/cncr.27968 PMC3673539

