# Supplementary figures and images for: The Utility of Sentinel Lymph Node Biopsy in Elderly Patients with Melanoma
Source: Ann Surg Oncol. 2024 Jul 22;31(12):8230–9. doi: 10.1245/s10434-024-15684-0 (PMC11467064; doi:10.1245/s10434-024-15684-0)

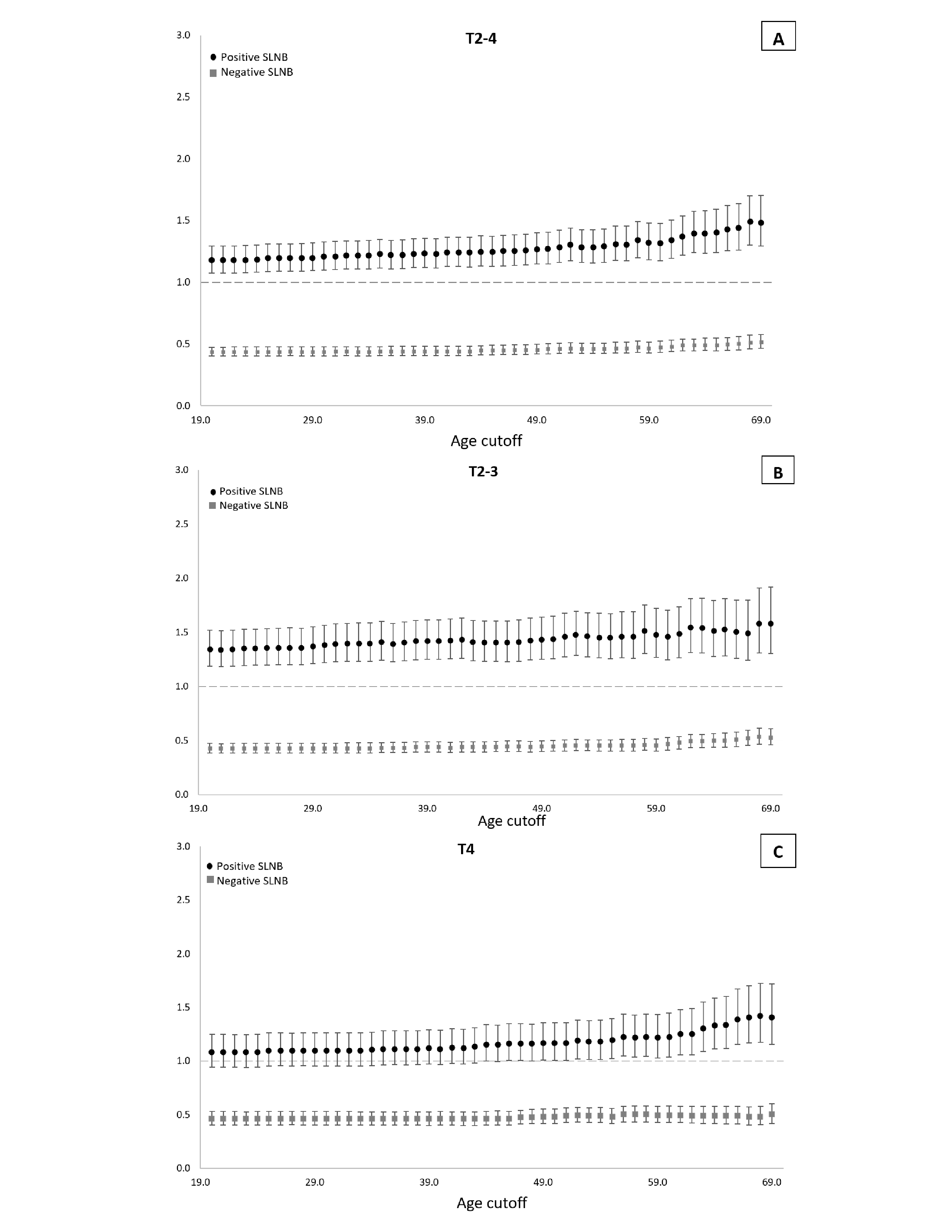

Supplement: Supplementary file 1 — Supplementary file1 (TIFF 260 kb) [file 10434_2024_15684_MOESM1_ESM.tiff]
