# Supplementary material for: The Utility of Sentinel Lymph Node Biopsy in Elderly Patients with Melanoma
Source: Ann Surg Oncol. 2024 Jul 22;31(12):8230–9. doi: 10.1245/s10434-024-15684-0 (PMC11467064; doi:10.1245/s10434-024-15684-0)
Supplement: Supplementary file 2 — Supplementary file2 (DOCX 17 kb) [file 10434_2024_15684_MOESM2_ESM.docx]

**Supplemental Table 1:** Multivariable Cox proportional hazard model for melanoma specific survival (SEER; 2010-2019) among patient ≥ 70 years olds diagnosed with clinically non-metastatic melanomas >1.0mm in Breslow thickness

|  | **Hazard ratio** | **p-value** | **95% CI** |
| --- | --- | --- | --- |
| **Sentinel lymph node biopsy (ref= not performed)** |  |  |  |
| **Sentinel lymph node negative** | 0.59 | <0.001 | 0.53-0.67 |
| **Sentinel lymph node positive** | 1.71 | <0.001 | 1.47-1.98 |
| **Age (ref= 70-79)** |  |  |  |
| **80-89** | 1.32 | <0.001 | 1.19-1.47 |
| **≥90** | 1.70 | <0.001 | 1.43-2.02 |
| **Sex (ref= male)** |  |  |  |
| **Female** | 0.83 | 0.001 | 0.74-0.92 |
| **Race (ref= non-Hispanic White)** |  |  |  |
| **Others** | 1.07 | 0.497 | 0.88-1.32 |
| **Tumor location (ref= trunk)** |  |  |  |
| **Head/Neck** | 0.89 | 0.089 | 0.78-1.02 |
| **Upper limb/shoulder** | 0.72 | <0.001 | 0.62-0.83 |
| **Lower limb/hip** | 0.82 | 0.019 | 0.69-0.97 |
| **Others** | 0.34 | 0.123 | 0.08-1.35 |
| **Tumor subtype (ref= superficial spreading)** |  |  |  |
| **Nodular** | 1.16 | 0.060 | 0.99-1.37 |
| **Lentigo maligna** | 0.96 | 0.815 | 0.69-1.34 |
| **Acral lentiginous** | 1.57 | 0.003 | 1.17-2.11 |
| **Desmoplastic** | 0.84 | 0.256 | 0.62-1.13 |
| **Others** | 1.07 | 0.367 | 0.92-1.24 |
| **Breslow thickness (ref= >1.0-2.0)** |  |  |  |
| **>2.0-4.0** | 1.62 | <0.001 | 1.42-1.86 |
| **>4.0** | 2.66 | <0.001 | 2.31-3.06 |
| **Ulceration (ref= no)** |  |  |  |
| **Yes** | 1.58 | <0.001 | 1.42-1.76 |
| **Mitosis (ref= non-mitogenic)** |  |  |  |
| **1 mitotic figure/mm^2^** | 1.17 | 0.225 | 0.91-1.51 |
| **≥2 mitotic figures/mm^2^** | 1.38 | 0.003 | 1.12-1.71 |
| **Others/unknown** | 1.50 | 0.001 | 1.18-1.90 |
| CI: Confidence interval | | | |
